# Slower respiration rate is associated with higher self-reported well-being after wellness training

**DOI:** 10.1038/s41598-023-43176-w

**Published:** 2023-09-24

**Authors:** Tammi R. A. Kral, Helen Y. Weng, Vikramjit Mitra, Theodore P. Imhoff-Smith, Erdrin Azemi, Robin I. Goldman, Melissa A. Rosenkranz, Sarah Wu, Andrew Chen, Richard J. Davidson

**Affiliations:** 1 Healthy Minds Innovations, Inc., Madison, WI USA; 2 Center for Healthy Minds, University of Wisconsin–Madison, Madison, WI USA; 3 Apple, Cupertino, CA USA; 4 Neuroscience Training Program, University of Wisconsin–Madison, Madison, WI USA; 5 Department of Psychology, University of Wisconsin–Madison, Madison, WI USA; 6 Department of Psychiatry, University of Wisconsin–Madison, Madison, WI USA

**Keywords:** Respiration, Human behaviour

## Abstract

Mind–body interventions such as mindfulness-based stress reduction (MBSR) may improve well-being by increasing awareness and regulation of physiological and cognitive states. However, it is unclear how practice may alter long-term, baseline physiological processes, and whether these changes reflect improved well-being. Using respiration rate (RR), which can be sensitive to effects of meditation, and 3 aspects of self-reported well-being (psychological well-being [PWB], distress, and medical symptoms), we tested pre-registered hypotheses that: (1) Lower baseline RR (in a resting, non-meditative state) would be a physiological marker associated with well-being, (2) MBSR would decrease RR, and (3) Training-related decreases in RR would be associated with improved well-being. We recruited 245 adults (age range = 18–65, M = 42.4): experienced meditators (*n* = 42), and meditation-naïve participants randomized to MBSR (*n* = 72), active control (*n* = 41), or waitlist control (*n* = 66). Data were collected at pre-randomization, post-intervention (or waiting), and long-term follow-up. Lower baseline RR was associated with lower psychological distress among long-term meditators (p* = 0.03, *b* = 0.02, 95% CI [0.01, 0.03]), though not in non-meditators prior to training. MBSR decreased RR compared to waitlist (*p* = 0.02, Cohen’s *d* = − 0.41, 95% CI [− 0.78, − 0.06]), but not the active control. Decreased RR related to decreased medical symptoms, across all participants (p* = 0.02, *b* = 0.57, 95% CI [0.15, 0.98]). Post-training, lower RR was associated with higher PWB across training groups compared to waitlist (p* = 0.01, *b* = 0.06, 95% CI [0.02, 0.10]), though there were no significant differences in change in PWB between groups. This physiological marker may indicate higher physical and/or psychological well-being in those who engage in wellness practices.

## Introduction

Stress, anxiety, and psychological distress hinder flourishing, while contributing to suffering. Worldwide, anxiety and depression cost an estimated $1 trillion annually in reduced productivity^1^. The sheer magnitude of the mental health crisis precludes addressing it by adding providers^2^. Accordingly, many researchers and clinicians have advocated for a mobile health approach to mental health, particularly targeting preventative strategies^3,4^. This effort will require the development of better, mobile measures of well-being, including the incorporation of biomarkers and biosignals. In this study, we sought to identify a passive biomarker of self-reported well-being, with sensitivity to detect training-related changes.

Respiration rate (RR) has potential as a biomarker of well-being, as a fundamental biological process that can be both automatic/non-conscious or conscious and modifiable^5^. RR is altered by, and may be a marker for, *states* of well-being and distress, both psychological and physical. For example, rapid, irregular RR has been associated with experiences of stress, anxiety, and pain^5–7^. Slower RR may indicate states of calm and restoration—slowing RR to about 6 breaths/min results in greater subjective relaxation^8^ and can reduce blood pressure^9^. RR may contribute to greater psychological and physical well-being through its engagement with the parasympathetic nervous system^10^. Indeed, the popular book *Breath* reviews a plethora of evidence showing the benefits of slower breathing and documents the impact of practices such as chanting and prayer on slowing RR^11^. Further, respiratory system malfunction can produce physical breathing disorders including pulmonary diseases and sleep apnea. RR may also be an ideal biomarker due to its measurability using mobile devices such as wearables^12,13^, smart phones^14^, wrist-wearables^15^, chest bands^16^, and wireless home devices^17^.

RR is responsive to interventions that directly control the breath, and those that indirectly impact the breath (e.g., mindful attention to the breath). Brief behavioral interventions that manipulate breathing can produce transient improvements in mood and emotional reactivity^18–20^. Behavioral interventions can change RR even without explicit instructions to alter breathing. In mindfulness meditation, instructions direct participants to nonjudgmentally attend to breathing sensations, without instructions to alter their breathing. Nevertheless, RR slows during meditation practice in novices^21–23^ and experts^24,25^. Moreover, short-term meditation training shows similar benefits to mental health and emotional reactivity as interventions that directly manipulate breath^26,27^.

This body of research indicates that slower RR may be a biomarker of well-being *states*, where alterations in well-being states may change to become more stable in the long-term through interventions such as mindfulness meditation. We have previously shown associations between slower baseline RR (during an awake, resting, non-meditative state) and more hours of lifetime meditation practice in those with long-term training^28^, and long-term meditators in the current study had significantly slower respiration rate than non-meditators^29^. It may be unsurprising that mindfulness meditation was associated with decreased RR, given the common attentional focus on breathing during practice, and bringing attention to automatic behaviors often results in a slowing of that behavior^30^. However, it is unknown whether slower RR may be specific to interventions that explicitly instruct participants to attend to their breathing in a different way, or to any wellness intervention that may benefit the cardiovascular system in some way. Although slower baseline RR in long-term meditators suggests that changing state-level RR consistently through meditation practice may lead to more stable changes, these findings may be due to pre-existing differences^31^. To our knowledge, a direct test of whether baseline RR may be a biomarker of general well-being, and whether meditation practice may produce stable, long-term RR changes, has not been reported.

This study examined relationships between baseline RR, meditation training, and well-being. We used extant data from a 3-arm randomized, active- controlled trial (RCT) of Mindfulness Based Stress Reduction (MBSR) that included a cross-sectional investigation of long-term meditators. We pre-registered the following hypotheses on the Open Science Framework (OSF; https://doi.org/10.17605/OSF.IO/TM87R): (1) slower baseline RR will be associated with better self-reported physical and psychological well-being in cross-sectional data (i.e., prior to randomization), as assessed by psychological distress and well-being, and medical symptoms; (2) MBSR will be associated with reduced RR (from pre- to post-training), compared to controls; (3) the degree of slowing in RR will be associated with improvements in self-reported well-being. Given that heart rate and heart rate variability (HRV) may also be important indicators for stress and health^32^ and can be measured with wearables, we proposed exploratory analyses testing whether lower heart rate, or higher heart rate variability (HRV), were associated with higher self-reported well-being.

## Methods

### Study design

This study was part of a larger clinical trial that was registered with ClinicalTrials.gov (NCT02157766), and the current analysis plan was pre-registered on OSF (https://doi.org/10.17605/OSF.IO/TM87R). The aims and findings of the larger clinical trial are beyond the scope of the current report, but, in brief, sought to test the efficacy of MBSR on change in 3 main outcomes: functional neurodynamics of emotional responses, diurnal cortisol, and lung function (in participants with asthma). To date, the study team has published positive findings with regards to the third outcome, whereby MBSR was associated with improved asthma control and reduced levels of exhaled nitric oxide^33^. This study was conducted entirely at the Center for Healthy Minds at the University of Wisconsin–Madison, and with the affiliated non-profit organization, Healthy Minds Innovations (HMI). Data were collected at the Waisman Laboratory for Brain Imaging and Behavior at the University of Wisconsin–Madison at three sessions: pre-randomization (T1), post-training (T2), and long-term follow-up (T3), approximately 6 months after T2. All procedures contributing to this work comply with the ethical standards of the relevant national and institutional committees on human experimentation and with the Helsinki Declaration of 1975, as revised in 2008. The study was approved by University of Wisconsin–Madison's Institutional Review Board (protocol number 2014-0116). All participants provided written consent and were given monetary compensation for participating.

### Participants

Healthy adults (18–65 years old) were recruited from the Madison, WI area for a study on “health and well-being” or the “benefits of health wellness classes”. Recruitment materials included flyers, online advertisements, and advertisements in local media. Participants in the RCT arm of the study had no prior training or formal practice in meditation or other mind–body techniques, or expertise in physical activity, music, or nutrition (based on the active control condition). Exclusion criteria for all participants included: use of psychotropic or nervous system altering medication; current diagnosis of sleep disorder; psychiatric diagnosis in the past year; any history of bipolar or schizoaffective disorders; history of traumatic brain injury or seizures; medical conditions; contraindications for safely participating in study procedures (e.g., ferromagnetic implants for MRI). The RCT also included a subset of participants with asthma, to address aims of the larger clinical trial that are beyond the scope of the current report. We conducted sensitivity analysis by running each analysis both with, and without, the participants with asthma. For detailed inclusion and exclusion criteria for the asthma group, who were not excluded for taking psychotropic drugs or a depression diagnosis, see Higgins et al., (2022).

Experienced meditators had to meet the following additional inclusion criteria: minimum 5 years of daily practice (minimum weekly average practice = 200 min); lifetime minimum of 5 weeks of meditation practice on retreat; experience in Vipassana, concentration, and compassion/loving-kindness meditations. We recruited meditators from meditation centers in the United States of America and their mailing lists, and with flyers and advertisements in the Madison, Wisconsin area.

Following screening, 256 participants were enrolled (see Supplementary Figure S1 for a CONSORT diagram), including 42 meditators, 133 meditation-naïve adults without asthma, and 70 meditation-naïve adults with asthma, from whom we had usable respiration data (see below for exclusion criteria). Initial sample sizes were based on a power analysis for the core hypotheses of the larger clinical trial. Eighteen participants left the study after randomization, and prior to data collection at the second session (eight MBSR, one HEP, and nine waitlist). Following randomization, we had respiration data at both sessions for the following groups: 72 MBSR, 41 active controls, and 66 waitlist. Participants with asthma were only randomized to MBSR or waitlist, and not to the active control condition, due to the limited sample size for this group. See Table 1 for detailed demographic information.

**Table 1:** Demographic information for all participants, by group.

		LTM	T1 Non-As	T1 Asthma	T2 MBSR		T2 Waitlist		T2 HEP
					Non-As	Asthma	Non-As	Asthma	
Sample size		42	133	70	39	33	36	30	41
Age (years)	Mean	44.2	44.0	37.5	45.1	38.9	46.5	35.8	42.7
	Min, Max	26.5, 66.6	25.1, 65.8	18.6, 65.1	25.2, 64.5	18.8, 65.1	25.8, 65.0	19.1, 61.3	25.1, 65.8
	SD	11.8	12.6	13.1	13.6	13.9	12.3	11.5	12.3
Sex	Female	16	81	42	22	17	24	18	25
	Male	26	52	28	17	16	12	12	15
Race*	Asian	1	9	5	4	3	4	2	1
	Black	2	2	1	0	1	0	0	2
	Native American	1	4	0	1	0	1	0	1
	Pacific Islander	0	1	0	0	0	1	0	0
	White	37	119	64	35	30	32	27	37
Ethnicity*	Hispanic	4	7	3	2	1	1	2	3
	Non-Hispanic	34	124	66	37	32	35	27	37

*MBSR* Mindfulness Based Stress Reduction; *HEP* Health Enhancement Program active control; *LTM* long-term meditator; *Non-As*. adults without asthma; *Min* minimum; *Max* maximum; *SD* standard deviation.

*Some participants selected more than one race, and some declined to provide race and/ or ethnicity information.

### Random assignment and training

Meditation-naïve participants were randomized following pre-training data collection, to either MBSR, waitlist control, or the Health Enhancement Program (HEP) active control. Randomization used stratified block assignment to balance age and gender across groups. The waitlist control group had a waiting period and did not participate in training. The HEP group was included to control for aspects of MBSR that were un-related to mindfulness meditation practice, and which might be common to other wellness programs, to test the specificity of the impact of mindfulness meditation training in MBSR. Such non-specific factors include the following: expertise of the instructor, length of the intervention, and the group structure of courses. The use of a matched, active control group (HEP) ensures that differences between groups are due to the skills learned in the interventions, rather than these non-specific factors^34^. HEP was designed to match the MBSR class structure, and differed only in content, providing training in nutrition, music therapy, balance and agility, and aerobic exercise^34^. HEP and MBSR instructors were experienced and certified, and all classes were taught through the Integrative Medicine program at the University of Wisconsin–Madison School of Medicine and Public Health in Madison, Wisconsin. All study staff, except the participant coordinator and project manager, were blinded to group assignment during data collection and processing using blinded group indicators, and only the participant coordinator had access to decode the data.

### Physiological measures

All peripheral physiological data were collected during a concurrent functional magnetic resonance imaging (fMRI) scan, amplified using a BIOPAC MP-150 system, and digitized at 1000 Hz. Participants were asked to remain calm, still, and awake with eyes fixed on a cross at the center of their visual field during the fMRI scan that lasted 12 min, and physiological data were collected for the duration of the scan. Inductance plethysmograph, a well-validated, standard respiration measure^35–37^, was used to capture respiration signal, and recorded on a polygraphic input box, using an abdominally placed, pneumatic belt. Mean RR (breaths per minute) was processed using trough-to-trough measurements, visually inspected for artifacts, and calculated using publicly available Matlab software (https://github.com/jwielgosz/resp_tools). Heart rate was measured using pulse oximetry and processed using CMetX^38^ with an inter-beat-interval (IBI) series corrected for artifact and ectopic beats. We calculated high frequency HRV, including interpolation, using the standard processing procedures in CMetX^38^.

### Self-report measures

To understand whether RR could represent a general biomarker for well-being, we assessed well-being broadly in three domains, and quantified with 3 self-report measures: (1) Psychological distress from the Global Symptoms Index (GSI) of the Symptoms Checklist 90 item survey (SCL-90)^39^ (2) Physical distress from the Medical Symptoms Checklist (MSC)^40^ (3) Psychological well-being from Ryff’s Psychological Well-Being (PWB) scale^41^. The SCL-90 assesses a broad range of psychological distress including anxiety, depression, and interpersonal sensitivity. The MSC assesses a range of physical symptoms including respiratory, muscular, and gastrointestinal symptoms. The PWB measures psychological well-being in several domains such as self-acceptance, autonomy, purpose in life, and positive relations with others. Greater PWB has been linked to better outcomes in physical health and biological regulation, as well as predicting reduced risk for diseases such as stroke and myocardial infarction^42^.

### Analysis

We used linear regression with two-tailed tests, and covariates for age and sex in all analyses due to differences in respiratory systems and well-being based on these factors^43–45^. Tests of training-related change (i.e., pre- to post-training) used difference scores (T2-T1). We assessed hypothesis 1 by regressing pre-randomization, self-reported well-being onto RR in our primary analysis. For hypothesis 2, we used a 3-way analysis of variance (ANOVA) to test for group differences in change scores, whereby we regressed the change score for the corresponding outcome variable (e.g., RR), onto the Group factor. We conducted separate analysis to test for pre-training (T1) differences in any of the measures between groups in the RCT. To test hypothesis 3, we regressed change in self-reported well-being onto change in RR, e.g., as our primary measure of interest.

We used a false discovery rate (FDR) correction to control for multiple comparisons for each family of confirmatory tests (i.e., for testing relationships between RR and each of the 3 well-being surveys), where a family is defined based on each hypothesis, using the *p.adjust* function with method “fdr”^46^ in the stats package version 4.3.0 in R Statistics version 4.1.0^47^. Results were determined to be statistically significant based on a threshold of p* < 0.05, where p* denotes the corrected *p*-value﻿. We excluded influential outliers based on Cook's D and excluded any points beyond the threshold of 4/(N-P), where N was the sample size, and P was the number of parameters in the model. The number of excluded cases due to influence based on Cook's D ranged from 0 to 14, or a maximum of 6% of the data. Respiration and heart rate data with extreme artifacts (e.g., due to movement, signal drop-out, or excessive ectopic beats), and/or insufficient amount of data (e.g., less than 5 min) were excluded from analysis.

Analyses were first conducted with all participants, and then separately when excluding participants with asthma, and for long-term meditators alone (cross-sectional analysis only), given the potential for differences in RR and/or the relationship between RR and well-being between these groups. All reported effect sizes are unstandardized beta estimates, based on the full model controlling for age and sex. Analysis of RCT data were also followed up to test whether effects held for the MBSR group alone. Finally, we conducted an intent-to-treat (ITT) analysis to confirm that post-randomization subject dropout did not bias the results, by including all participants who were randomized in the RCT analysis. The ITT analysis used a linear mixed effects model (LMEM) with restricted maximum likelihood (REML) estimation, implemented in R with the *lmer* function of the lme4 package version 1.1-33^48^, and the *summary* function of the lmerTest package version 3.1-3^49^.

## Results

### Deviations from pre-registration

The current study deviated from the pre-registration in the following ways. Exploratory analyses were conducted that were not pre-registered, including analysis of HRV, and these analyses are identified as “exploratory” in the text. We added an ITT analysis using linear mixed effects modeling on all cases as a sensitivity analysis of the RCT effects. The current report focuses on the relationship between respiration and global psychological and physical well-being and excludes investigation of hypotheses around the relationship between respiration and dispositional negativity, depression, and mood states.

### Overview and demographics

Descriptive statistics for all measures are presented in Table 2, and all tests controlled for age and sex. Older participants had significantly slower RR, such that a 1-year increase in age corresponded to .11 breaths/ minute decrease in RR (*p* < 0.01, 95% CI = [− .15, − 0.08]). Participants with asthma were significantly younger than meditators (*p* = 0.01, *b* = 6.46, 95% CI = [− 11.31, − 1.62]) and non-meditators without asthma (*p* < 0.01, *b* = − 7.64, 95% CI = [− 11.31, − 3.96]). There was no difference in pre-training, baseline RR in non-meditators with and without asthma (*p* = 0.55, *b* = 0.30, 95% CI = [− 0.68, 1.27]).

**Table 2:** Descriptive statistics for all measures, by group and session.

Measure	Session	MBSR		Waitlist		HEP	LTM
		Non-As	Asthma	Non-As	Asthma	Non-As	Non-As
RR	T1 mean	13.86	14.88	14.85	15.24	14.20	11.51
	T1 SD	3.48	3.44	3.62	4.09	3.90	4.13
	T2 mean	13.42	14.16	14.81	15.44	14.43	–
	T2 SD	3.27	3.43	3.75	3.92	3.75	–
HR	T1 mean	66.52	68.30	66.27	70.23	66.67	69.88
	T1 SD	8.92	8.74	6.64	9.96	10.97	11.53
	T2 mean	64.94	66.16	64.87	68.74	63.54	–
	T2 SD	9.68	9.75	8.35	11.28	8.41	–
Log HRV	T1 mean	7.72	7.68	7.58	7.54	7.70	7.81
	T1 SD	0.80	0.71	0.76	0.78	0.95	0.96
	T2 mean	7.86	7.88	7.68	7.78	7.92	–
	T2 SD	0.82	0.94	0.83	0.83	0.96	–
SCL90	T1 mean	0.29	0.42	0.27	0.38	0.22	0.24
	T1 SD	0.22	0.32	0.20	0.27	0.17	0.21
	T2 mean	0.25	0.36	0.29	0.38	0.25	–
	T2 SD	0.23	0.27	0.24	0.27	0.24	–
MSC	T1 mean	8.74	14.03	8.21	13.52	5.10	7.60
	T1 SD	7.76	9.01	7.37	9.47	5.36	8.06
	T2 mean	8.65	12.29	8.87	12.78	6.50	–
	T2 SD	7.73	8.92	8.60	9.49	7.47	–
PWB	T1 mean	193.95	191.41	198.09	186.44	204.49	209.90
	T1 SD	26.17	26.55	23.74	24.47	27.23	18.56
	T2 mean	195.02	193.38	193.36	188.03	202.63	–
	T2 SD	22.43	28.13	28.1	30.81	31.11	–

*MBSR* Mindfulness Based Stress Reduction; *HEP* Health Enhancement Program active control; *LTM* long-term meditator; *Non-As*. adults without asthma; *RR* respiration rate; *HR* heart rate; *HRV* heart rate variability; *SCL90* Symptoms Checklist 90; *MSC* Medical Symptoms Checklist; *PWB* Psychological Well-being; *T1* time 1 pre-training session; *T2* time 2 post-training session; *SD* standard deviation.

There was no effect of sex on RR (*p* = 0.70, *b* = 0.19, 95% CI = [− 0.78, 1.16]*).* Meditators had significantly more male participants than non-meditators without asthma (*p* = 0.02, *b* = 0.23, 95% CI = [0.06, 0.40]) and with asthma (*p* = 0.01, *b* = 0.22, CI = [0.04, 0.41]). There were no differences in age or sex of participants in the MBSR group compared to control groups in the RCT (*p* > 0.10). Detailed results for all statistical analyses in this section are provided in Supplementary Table 1.

### Confirmatory cross-sectional analysis: associations between RR and well-being

We tested whether slower RR may be a general physiological marker of self-reported well-being. When testing across all participants, there were no significant associations between RR and distress (p* = 0.07, *b* = 0.01, 95% CI = [0.00, 0.02]), medical symptoms (p* = 0.54, *b* = 0.90, 95% CI = [− 0.19, 0.37]), or psychological well-being (p* = 0.32, *b* = − 0.50, 95% CI = [− 1.28, 0.29]).

**Figure 1:**
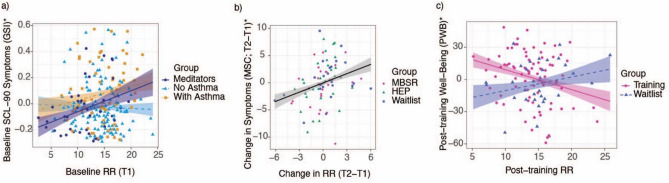
Associations between baseline respiration rate (RR) and self-reported well-being. (**a**) Slower baseline RR was associated with significantly less distress among meditators (p* = 0.02). (**b**) Larger reductions in RR were associated with larger improvements on the Medical Symptoms Checklist (MSC), across all non-asthmatic adults (p* = 0.02). (**c**) MBSR and active control participants had significantly stronger relationships between RR and self-reported psychological well-being, post-training, compared to waitlist (*p* = 0.03). All analyses controlled for age and gender, and data points are displayed after adjustment for the covariates. Confidence intervals represent 1 standard error from the point estimate of the mean. T1 = time 1 (pre-randomization); T2 = time 2 (post-training); HEP = Health Enhancement Program active control; MSC = Medical Symptoms Checklist; SCL = Symptoms Checklist.

Among long-term meditators alone, slower RR was associated with significantly less psychological distress (p* = 0.02, *b* = 0.02, 95% CI = [0.01, 0.03]; SCL90; dark blue line, Fig. 1a) and higher psychological well-being (p* = 0.04, *b* = − 1.43, 95% CI = [− 2.70, − 0.15]). For every one breath per minute slower respiration rate, meditators had 0.02 lower SCL-90 distress scores, and 1.43 higher well-being scores, controlling for age and sex. There was no significant relationship between RR and medical symptoms (p* = 0.06, *b* = 0.45, 95% CI = [− 0.02, 0.91]; MSC). We conducted additional analyses to test the relationships between RR and well-being using log transformed SCL90 and MSC (separately), to correct for left skew (due to recruitment of a generally healthy adult sample), and both effects were significant among meditators (SCL90: p* = 0.02, *b* = 3.26, 95% CI = [0.65, 5.87]; MSC: p* = 0.02, *b* = 3.74, 95% CI = [0.70, 6.77]). Detailed results for all statistical analyses in this section are provided in Supplementary Table 1.

Given the positive association between RR and SCL90 global distress, we conducted post-hoc analyses to examine which 9 sub-scales and 2 other composite measures of the SCL90 were driving this effect, as described in our pre-registration. Detailed statistical results for SCL90 scales are presented in Supplementary Table 2. Across all participants, lower total symptoms, lower anger-hostility, and lower interpersonal sensitivity (e.g., “Feeling critical of others”), were significantly associated with lower RR, separately (total symptoms: p* = 0.02, *b* = 0.71, 95% CI = [0.25, 1.16]; anger-hostility: p* = 0.04, *b* = 0.01, 95% CI = [0.00, 0.02]; sensitivity: p* < 0.01, *b* = 0.02, CI = [0.01, 0.03]). For every 1 breath per minute slower RR, participants had 0.71 fewer total symptoms, 0.01 lower anger-hostility, and 0.02 lower interpersonal sensitivity distress symptoms. When testing separately among experienced meditators, higher depression, obsessive compulsions, and phobic anxiety were additionally associated with higher RR (depression: p* = 0.01, *b* = 0.03, 95% CI = [0.01, 0.05]; obsessive compulsion: p* = 0.01, *b* = 0.04, 95% CI = [0.01, 0.06]; phobic anxiety: p* = 0.03, *b* = 0.01, 95% CI = [0.00, 0.01]).

### Confirmatory RCT analysis: MBSR-related changes

**Figure 2:**
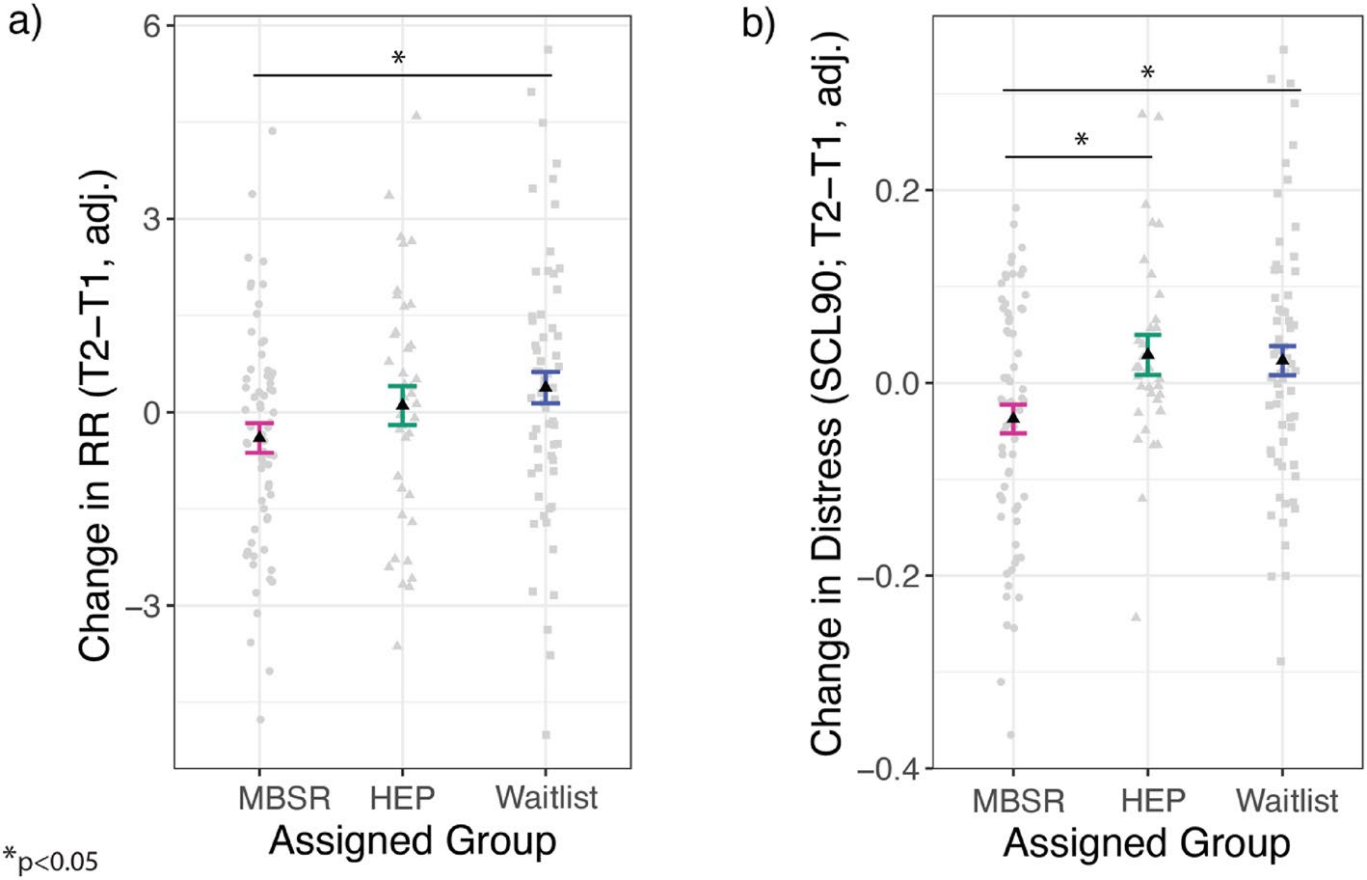
Mindfulness Based Stress Reduction- (MBSR) related changes in respiration rate (RR) and self-reported well-being. (**a**) Participants in MBSR had reduced RR from pre- to post-training compared to Waitlist (*p* = 0.01), but not the Health Enhancement Program (HEP) active control (*p* = 0.20). (**b**) MBSR participants had reduced distress on the Symptom Checklist (SCL) 90 Global Severity Index from pre- to post-training, compared to waitlist (*p* = 0.01) and HEP (*p* = 0.02). All analyses controlled for age and gender, and data points are displayed after adjustment for the covariates. Confidence intervals represent 1 standard error from the point estimate of the mean. T1 = time 1 (pre-randomization); T2 = time 2 (post-training); HEP = Health Enhancement Program active control; SCL = Symptoms Checklist.

MBSR was associated with a significantly larger reduction in baseline RR from pre- to post-training (T2-T1) compared to waitlist (*p* = 0.01, *b* = 0.88, 95% CI [0.23, 1.54]), but not HEP (*p* = 0.20, *b* = 0.48, 95% CI [− 0.25, 1.21]) (Fig. 2a). MBSR participants reduced their RR by 0.88 breaths per minute, relative to waitlist controls. Sensitivity analysis using LMEM produced consistent results to our primary analysis of RR change scores, whereby there was a significant interaction of Group by Session on RR (MBSR vs waitlist: *p* = 0.04, *b* = 0.84, 95% CI [0.06, 1.62]; MBSR vs HEP: *p* = 0.07, *b* = 0.85, 95% CI [− 0.05, 1.75]). The group difference between MBSR and waitlist was not maintained at long-term follow-up (*p* = 0.15, *b* = 0.50, 95% CI [− 0.19, 1.19). There was no difference in change in RR between HEP and waitlist (*p* = 0.29, *b* = 0.40, 95% CI [− 0.35, 1.15]), and there were no differences between groups when limiting the analysis to the sub-sample of participants without asthma *(p* > 0.10)*.* There were no differences in RR between RCT intervention groups at T1 (*p* > 0.10). Detailed results for all statistical analyses in this section are provided in Supplementary Table 1.

### Exploratory RCT analysis: MBSR-related changes in self-reported well-being

We tested whether MBSR-related changes in RR were accompanied by improvements in self-reported well-being, compared to controls. MBSR significantly reduced psychological distress (SCL-90) more than waitlist (*p* = 0.01, *b* = 0.06, 95% CI = [0.02, 0.10]) or HEP (*p* = 0.02, *b* = 0.06, 95% CI = [0.01, 0.11]; Fig. 2b). MBSR participants had 0.06 less symptoms than waitlist and active control groups, from pre- to post-training, which remained significant when restricting analysis to participants without asthma.

While there was no difference in SCL-90 scores between MBSR and waitlist at T1 (*p* = 0.54, *b* = − 0.03, 95% CI = [− 0.11, 0.05]), MBSR participants had 0.11 higher SCL90 distress scores compared to HEP at T1 (*p* = 0.02, *b* = − 0.11, 95% CI = [− 0.22, − 0.03]), pre-randomization. We re-ran the analysis of intervention effects on change in SCL90 scores controlling for T1 scores, and separately, in a group by time repeated measures LMEM (regressing SCL-90 scores on the interaction of group and session, including by-subject random effects), to ensure that the results were not due to the chosen modelling strategy, and the results remained significant (*p* < 0.05). There were no differences in change in psychological well-being (PWB) or change in medical symptoms (MSC) for MBSR and either control group, with or without participants with asthma (*p* > 0.10). Detailed results for all statistical analyses in this section are provided in Supplementary Table 3.

### Confirmatory RCT analysis: associations between RR and well-being

We examined whether reductions in RR were associated with improvements with self-reported well-being, across all participants in the RCT, given that we hypothesized RR as a potential general biomarker for well-being. Thus, we expected reductions in RR to be associated with improved self-reported well-being, regardless of treatment condition. When including participants with asthma, there were no significant associations between changes in RR and self-reported well-being across the entire sample (SCL90: p* = 0.37, *b* = 0.01, 95% CI = [0.01, 0.01]; PWB: p* = 0.97, *b* = -0.02, 95% CI = [− 0.73, 0.70]; MSC: p* = 0.37, *b* = 0.20, 95% CI = [− 0.14, 0.53]). Among participants without asthma, reduced RR was associated with significantly reduced symptoms on the MSC (p* = 0.02, *b* = 0.57, 95% CI = [0.15, 0.98]; Fig. 1b). For every one breath per minute reduction in RR, participants had 0.57 lower scores on the MSC (controlling for age and sex). Within the MBSR group, the relationships between change in RR and change in symptoms was non-significant (MSC: p* = 0.17, *b* = 0.08, 95% CI = [− 0.06, 0.23]). There was no relationship between change in RR and change in distress on SCL90 (p* = 0.05, *b* = 0.01, 95% CI = [0.00, 0.02]), or in PWB (p* = 0.44, *b* = − 0.35, 95% CI = [− 1.27, 0.56]), across RCT groups in participants without asthma.

To examine whether baseline RR could be a physiological marker of self-reported well-being following wellness training, we tested whether slower RR was associated with higher self-reported well-being following wellness training, during the second session (T2). Since the MBSR and HEP groups did not significantly differ in change in RR (pre- to post-intervention), and since HEP was designed as a rigorous active control for mindfulness-related aspects of MBSR, and as we previously reported that HEP improved self-reported “mindfulness”^50^, we combined across MBSR and HEP for this analysis. Thus, we tested whether RR could index a more general signal for self-reported well-being, in the context of well-being interventions more broadly than mindfulness-based practices.

Among adults without asthma following training, slower baseline RR for participants who took MBSR or HEP (combined) was associated with significantly higher psychological well-being (*p* = 0.03, *b* = 2.98, 95% CI = [0.33, 5.64]; PWB; Fig. 1c), compared to waitlist. The relationship with post-training RR and post-training psychological well-being was significant within MBSR and HEP (combined) (*p* = 0.03, *b* = − 1.83, 95% CI = [− 3.50, − 0.16]). One breath per minute slower RR, following training, was associated with a 1.83 higher psychological well-being score among MBSR and HEP participants, controlling for age and sex. The relationship between post-training RR and well-being was not significantly different between waitlist and MBSR alone** (p** = 0.62, *b* = 0.02, 95% CI = [− 0.08, 0.05]).

Among participants without asthma, there was no post-training association between RR and distress (*p* = 0.58, *b* = 0.00, 95% CI = [− 0.01, 0.02]**;** SCL90) or medical symptoms (*p* = 0.12, *b* = 0.36, 95% CI = [− 0.01, 0.82]), nor were there significant differences between training and waitlist groups in this relationship across training groups (SCL90: *p* = 0.63, *b* = − 0.01, 95% CI = [− 0.03, 0.01]**;** MSC: *p* = 0.06, *b* = 0.91, 95% CI = [− 1.85, 0.04]). There were no significant differences between training and waitlist groups in the relationships between RR and self-reported well-being at post-training when including participants with asthma *(p* > 0.10)*.* Detailed results for all statistical analyses in this section are provided in Supplementary Table 3.

### Exploratory analysis

There were no significant relationships between baseline heart rate and distress (p* = 0.59, *b* = 0.00, 95% CI = [0.00, 0.00]), psychological well-being (p* = 0.59, *b* = − 0.13, 95% CI = [− 0.44, 0.19]) or medical symptoms (p* = 0.59, *b* = 0.05, 95% CI = [− 0.06, 0.16]), when looking across all participants (pre-randomization for non-meditators). These relationships remained non-significant when excluding participants with asthma and when looking only among meditators (*p* > 0.10). There were no differences in change in HR between MBSR and waitlist (p* = 0.24, *b* = 0.04, 95% CI = [− 1.78, 1.86) or HEP (p* = 0.97, *b* = − 1.23, 95% CI = [− 3.28, 0.82]), and there were no relationships between change in HR and change in distress (p* = 0.67, *b* = 0.00, 95% CI = [0.00, 0.00]), PWB (p* = 0.52, *b* = 0.14, 95% CI = [− 0.15, 0.43]) or medical symptoms (p* = 0.52, *b* = − 0.06, 95% CI = [− 0.19, 0.06]), when looking across all RCT participants. These effects remained non-significant when excluding participants with asthma from analysis. See Supplementary Table 4 for detailed statistical results for tests of HR.

Across all participants, higher baseline HRV was associated with higher psychological well-being (p* < 0.01, *b* = 7.25, 95% CI = [3.43, 11.06]) and less distress (p* = 0.01, *b* = − 0.06, 95% CI = [− 0.10, − 0.03]), prior to randomization**.** These relationships were not significant when restricting the sample to meditators (p* > 0.05). There was no relationship between baseline HRV and medical symptoms (p* = 0.18, *b* = − 0.90, 95% CI = [− 2.21, 0.43]).

There were no significant relationships between change in HRV and change in self-reported well-being when testing across the full sample from the RCT, including participants with asthma (SCL90: p* = 0.42, *b* = − 0.02, 95% CI = [− 0.02, 0.02]; PWB: p* = 0.98, *b* = 0.04, 95% CI = [− 3.19, 3.27]; MSC: p* = 0.42, *b* = − 0.80, 95% CI = [− 2.20, 0.61]), nor when restricting analysis to participants without asthma.

While there were no significant differences in change in HRV between MBSR and waitlist or HEP, following the training period (WL: *p* = 0.93, *b* = 0.01, 95% CI = [− 0.26, 0.10]; HEP: *p* = 0.41, *b* = − 0.08, 95% CI = [− 0.15, 0.17]), reduced RR was associated with increased HRV, across all participants from pre- to post-intervention (*p* = 0.01, *b* = − 0.04, CI = [− 0.07, − 0.01]), and this relationship remained significant when excluding participants with asthma (*p* < 0.05). Detailed statistical results for all tests of HRV are presented in Supplementary Table 5.

## Discussion

This study provides initial evidence for RR as a potential biomarker of self-reported well-being in the context of wellness training. We found a similar pattern in novice groups following short-term training, and in meditators with a long-term, lifetime practice on the order of 9900 total hours of practice—slower RR was associated with better self-reported well-being in terms of mental and physical health. In addition to the positive results in support of our hypotheses, we have also reported many null findings for consideration. There were no significant relationships between RR and self-reported well-being prior to randomization (T1) among non-meditators, however, short-term MBSR training reduced RR relative to the waitlist condition. Moreover, larger reductions in RR were correlated with larger improvements in medical symptoms, across all participants.

In exploratory analysis, we found that post-training associations between slower RR and better self-reported psychological well-being were present among novices who were trained in either MBSR or HEP, compared to waitlist. As there were also no differences in RR change between MBSR and HEP from pre- to post-training, these effects may not be limited to mindfulness-based training and may apply to other types of wellness training. However, participants in HEP did not differ from waitlist in changes in RR or self-reported well-being.

The group difference in change in RR between MBSR and waitlist was not maintained at long-term follow-up. This is consistent with our prior reporting on a subset of the same participants, showing that changes in resting state functional brain connectivity were also not maintained at follow-up^51^. Short-term mindfulness practice with MBSR may produce temporary reductions in baseline RR, which may become more stable with long-term practice.

Mindfulness meditation is one type of wellness training that is well-suited to maximize the benefits of RR-related associations with, and improvements in, well-being. Slowing in respiration may in part reflect the impact of bringing awareness to breathing, and slower breathing may be associated with changes in other biological systems (e.g., vagally mediated processes) that mediate reductions in symptoms and improvements in well-being. Studies suggest there are greater health benefits to mindfulness practice than guided slow breathing alone^9^ due to interoceptive and emotion regulation skills^10^, and future studies should further examine how these mechanisms contribute to well-being.

While the RCT aspect of the current study provided evidence of the efficacy of MBSR for reducing distress and RR, there were no significant improvements in the psychological well-being compared to control groups, with Ryff's widely used Psychological Well-being scales. This is consistent with preliminary results of recent research from an independent study, showing a lack of improvement in the same scales of psychological well-being with both an 8-week and an 18-week meditation training program, though this work remains to be peer reviewed^52^. Yet, both training groups increased in the autonomy subscale, and, thus, some aspects of psychological well-being may be responsive to mindfulness or other types of wellness training. We also previously reported more general improvements in mindfulness among both MBSR and HEP participants from the current study, as assessed with the Five Facet Mindfulness Questionnaire^53^. Thus, one possibility is that MBSR targets aspects of well-being that aren’t well-captured by Ryff's Psychological Well-being scales.

Additional sources of objective and subjective well-being are needed to understand whether slower RR may be a physiological marker of well-being for populations apart from those who engaged in mind–body training, as the lack of a pre-training relationship may be due to differences in providing accurate self-reports. Mind–body training such as mindfulness may improve interoceptive awareness, or subjective attention and reporting of bodily states^54^, which may have contributed to the significant relationships between RR and self-reported well-being.

Our exploratory analyses provided strong evidence that HRV may be a more general biomarker for self-reported well-being, though it was less responsive to meditation training. Higher HRV showed strong associations with all measures of well-being in this study, in line with prior research^32,55,56^. Confirmatory research is warranted to assess whether, and how, higher HRV may be a biomarker for self-reported well-being. Heart rate failed to show any significant associations with self-reported well-being in exploratory analyses, and was unresponsive to mindfulness training, as was seen in previous research where mindfulness of pain decreased RR during practice, but had no effect on HR, in experienced meditators^57^.

The aims and hypotheses of this study were agnostic to asthma diagnosis, though participants with asthma were recruited as a separate group, to address hypotheses of the larger, parent study for which this extant data was originally collected. We planned analyses with and without inclusion of these participants, given that including individuals with asthma may lead to differential results. In fact, group differences in RR following MBSR were only significant with the larger sample, including participants with asthma, while post-training relationships between RR and self-reported psychological well-being were only present when participants with asthma were excluded. Future research could explore the impact of asthma, and other respiratory conditions, on the relationship between RR, well-being, and wellness training.

This study was limited by a lack of diversity in the sample population, and in the method of characterizing respiration, due to the use of a convenience sample from extant data. The larger clinical trial sampled from a very healthy, largely white, midwestern USA, adult population—owing to the aims of the clinical trial (i.e., exclusion criteria to reduce confounds, the geographical location of the study team, and available resources). Future research must evaluate whether associations between slower RR and higher well-being, and their sensitivity to change, generalize to a broader, more diverse population. Replicating these results with a larger sample, and with physiological data collected over more than a single time window, will help address whether the effects are stable, and to test the veracity of the results reported here. In order to directly test the transfer of state effects to more stable, long-term changes in RR and well-being, future research should aim to collect physiological measures prior to meditation experience, during practice, and at different points following meditation practice, in the same participants.

The physiological data in this study were collected during an MRI scan, which is very different from the typical context of daily living, and participants were not blind to the timing and presence of physiological data collection. Thus, future research should aim to replicate these findings in real-world contexts, with wearable sensors that are covert or less obtrusive, and would allow double-blind testing of physiological signals, and where environmental effects on respiration could also be examined. In addition, an important extension of this work is to examine a more comprehensive set of respiration measures, including depth, nasal versus oral, and relative proportion of abdominal to thoracic and inhalation to exhalation. It is critical to carefully consider the complex interrelation of different aspects of respiration psychophysiology and wellness training, to optimize outcomes, and to avoid or mitigate potential risks.

In conclusion, this research provides support for RR as a biomarker for self-reported well-being in the context of wellness training, and preliminary evidence for HRV as a more general, but less plastic, well-being biomarker. Passive, physiological measures have potential to provide assessments of well-being, and training-related plasticity, at scale.

## Supplementary Information

Supplementary Figure S1.

Supplementary Table S1.

Supplementary Table S2.

Supplementary Table S3.

Supplementary Table S4.

Supplementary Table S5.

## Data Availability

Anonymized data and analysis code are available on OSF (https://osf.io/f25j9/).
