# Correction: Slower respiration rate is associated with higher self-reported well-being after wellness training

**DOI:** 10.1038/s41598-026-35107-2

**Published:** 2026-01-29

**Authors:** Tammi R. A. Kral, Helen Y. Weng, Vikramjit Mitra, Theodore P. Imhoff‑Smith, Erdrin Azemi, Robin I. Goldman, Melissa A. Rosenkranz, Sarah Wu, Andrew Chen, Richard J. Davidson

**Affiliations:** 1 Healthy Minds Innovations, Inc., Madison, WI USA; 2 Center for Healthy Minds, University of Wisconsin–Madison, Madison, WI USA; 3 Apple, Cupertino, CA USA; 4 Neuroscience Training Program, University of Wisconsin–Madison, Madison, WI USA; 5 Department of Psychology, University of Wisconsin–Madison, Madison, WI USA; 6 Department of Psychiatry, University of Wisconsin–Madison, Madison, WI USA

Correction to: *Scientific Reports* 10.1038/s41598-023-43176-w, published online 24 September 2023

The original version of this Article contained an error in the Results section under the subheading ‘Overview and demographics’, where the effect sizes for the associations between respiration rate and demographic factors (age and sex) were incorrectly reported.

As a result,

“Older participants had significantly slower RR, such that a 1 year increase in age corresponded to 1.35 breaths/minute decrease in RR (p < 0.01, 95% CI=[− 1.73, − 0.96]). Participants with asthma were significantly younger than meditators (p=0.01, b=6.46, 95% CI=[− 11.31, − 1.62]) and non-meditators without asthma (p<0.01, b=− 7.64, 95% CI=[− 11.31, − 3.96]). There was no difference in pre-training, baseline RR in non-meditators with and without asthma (p=0.55, b=0.30, 95% CI=[− 0.68, 1.27]).

There was no effect of sex on RR (p=0.94, b=0.00, 95% CI=[− 0.02, 0.02]).”

now reads:

“Older participants had significantly slower RR, such that a 1-year increase in age corresponded to .11 breaths/minute decrease in RR (p < 0.01, 95% CI=[− .15, − 0.08]). Participants with asthma were significantly younger than meditators (p=0.01, b=6.46, 95% CI=[− 11.31, − 1.62]) and non-meditators without asthma (p<0.01, b=− 7.64, 95% CI=[− 11.31, − 3.96]). There was no difference in pre-training, baseline RR in non-meditators with and without asthma (p=0.55, b=0.30, 95% CI=[− 0.68, 1.27]).

There was no effect of sex on RR (p=0.70, b=0.19, 95% CI=[− 0.78, 1.16]).”

The original Article has been corrected.

