# Supplementary material for: Slower respiration rate is associated with higher self-reported well-being after wellness training
Source: Sci Rep. 2023 Sep 24;13:15953. doi: 10.1038/s41598-023-43176-w (PMC10518325; doi:10.1038/s41598-023-43176-w)
Supplement: Supplementary file 1 — Supplementary Figure S1. [file 41598_2023_43176_MOESM1_ESM.pdf]

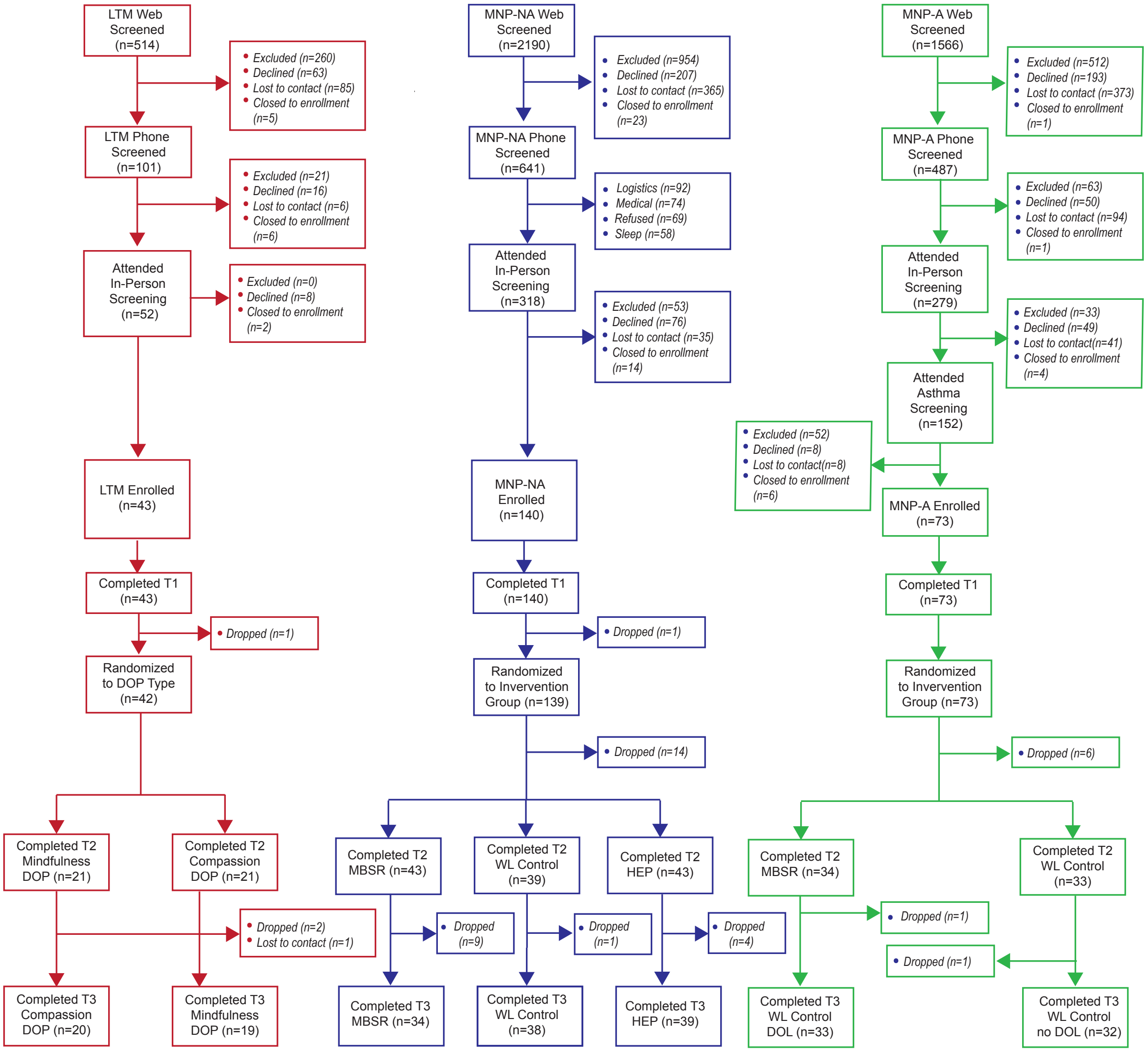

Fig. S1. CONSORT diagram. This study includes data from the randomized controlled trial of non-asthmatic, meditation-naïve participants (MNP-NA), shown in blue in the middle. Participants completed a baseline lab visit (T1) prior to randomization to either Mindfulness-Based Stress Reduction (MBSR), the Health Enhancement Program (HEP) active control intervention, or a waitlist control (WL) group. Participants completed a post-intervention (T2) lab visit, and a third lab visit for long-term follow-up (T3). This figure was originally published in Kral *et al.*, 2022, *Science Advances* (doi: 10.1126/sciadv.abk3316).
