# Supplementary material for: Slower respiration rate is associated with higher self-reported well-being after wellness training
Source: Sci Rep. 2023 Sep 24;13:15953. doi: 10.1038/s41598-023-43176-w (PMC10518325; doi:10.1038/s41598-023-43176-w)
Supplement: Supplementary file 2 — Supplementary Table S1. [file 41598_2023_43176_MOESM2_ESM.docx]

Table S1. Detailed statistical results for tests of respiration rate, demographics.

| Model* (type) | Sample | Contrast | *p* | *b* | CI |
| --- | --- | --- | --- | --- | --- |
| T1 RR ~ Age | All subjects | - | <0.01 | -1.35 | -1.73, -0.96 |
| Age ~ Group | All subjects | Meditator - MNP with asthma | 0.01 | -6.46 | -11.31, -1.62 |
|  |  | Meditator - MNP no asthma | 0.98 | 0.06 | -4.34, 4.46 |
|  | MNP | No Asthma - Asthma | <0.01 | -7.64 | -11.31, -3.96 |
| T1 RR ~ Sex | All subjects | - | 0.94 | 0.00 | -0.02, 0.02 |
| Sex ~ Group | All Subjects | Meditator - MNP with asthma | 0.02 | 0.22 | 0.04, 0.41 |
|  |  | Meditator - MNP no asthma | 0.01 | 0.23 | 0.06, 0.40 |
|  | MNP | MNP no asthma - with asthma | 0.98 | 0.00 | -0.14, 0.14 |
| T1 RR ~ T1 SCL90 (H1) | All subjects | - | 0.07 | 0.01 | 0.00, 0.02 |
|  | MNP no asthma | - | 0.95 | 0.00 | -0.01, 0.01 |
|  | Meditators | - | 0.02 | 0.02 | 0.01, 0.03 |
| T1 RR ~ T1 SCL90 x Group | All subjects | Meditators - MNP no asthma | 0.05 | -0.02 | -0.04, 0.00 |
|  |  | Meditators - MNP with asthma | 0.69 | 0.00 | -0.03, 0.02 |
| T1 RR ~ T1 PWB (H1) | All subjects | - | 0.32 | -0.50 | -1.28, 0.29 |
|  | MNP no asthma | - | 0.91 | 0.59 | -0.59, 1.78 |
|  | Meditators | - | 0.04 | -1.43 | -2.70, -0.15 |
| T1 RR ~ T1 MSC (H1) | All subjects | - | 0.54 | 0.09 | -0.19, 0.37 |
|  | MNP no asthma | - | 0.91 | -0.10 | -0.48, 0.28 |
|  | Meditators | - | 0.06 | 0.45 | -0.02, 0.91 |
| T1 RR ~ Log T1 SCL90 | Meditators | - | 0.02 | 3.26 | 0.65, 5.87 |
| T1 RR ~ Log T1 MSC | Meditators | - | 0.02 | 3.74 | 0.70, 6.77 |
| Delta RR ~ Group (Pre/Post; LM; H2) | All MNP | MBSR - WL | 0.01 | 0.88 | 0.23, 1.54 |
|  |  | MBSR - HEP | 0.20 | 0.48 | -0.25, 1.21 |
|  |  | HEP - WL | 0.29 | 0.40 | -0.35, 1.15 |
|  | No asthma | MBSR - WL | 0.23 | 0.58 | -0.37, 1.53 |
|  |  | MBSR - HEP | 0.80 | 0.11 | -0.79, 1.01 |
| Delta RR ~ Group (Pre/Follow-up) | All MNP | MBSR - WL | 0.15 | 0.50 | -0.19, 1.19 |
| RR ~ Group x Time  (LMEM; ITT) | All MNP | MBSR - WL x T2 - T1 | 0.04 | *b* = 0.84 | 0.06, 1.62 |
|  |  | MBSR - HEP x T2 - T1 | 0.07 | *b* = 0.85 | -0.05, 1.75 |
| T1 RR ~ Group | All MNP | MBSR - WL | 0.42 | 0.48 | -0.64, 1.86 |
|  |  | MBSR - HEP | 0.94 | -0.05 | -1.53, 1.33 |
|  |  | MNP with asthma - no asthma | 0.55 | 0.30 | -0.68, 1.27 |

*All models included covariates for age and sex.
Note: CI = confidence interval (of effect size estimate); RR = respiration rate; T = time-point; PWB = Psychological Well-being; H = (confirmatory) hypothesis; SCL90 = Symptoms Checklist 90; MSC = Medical Symptoms Checklist; LM = linear model; LMEM = linear mixed effects model; ITT = intent-to-treat; MNP = meditation=naïve participant; WL = waitlist; HEP = health enhancement program (active control); Pre = pre-training period; Post = post-training period; Follow-up = 6 months post-training period
