# Supplementary material for: Slower respiration rate is associated with higher self-reported well-being after wellness training
Source: Sci Rep. 2023 Sep 24;13:15953. doi: 10.1038/s41598-023-43176-w (PMC10518325; doi:10.1038/s41598-023-43176-w)
Supplement: Supplementary file 3 — Supplementary Table S2. [file 41598_2023_43176_MOESM3_ESM.docx]

Table S2. Detailed statistical results for tests of T1 respiration rate regressed on T1 distress scales.

| SCL90 scale | Sample | *p* | *b* | CI |
| --- | --- | --- | --- | --- |
| Anxiety | All subjects | 0.90 | 0.00 | -0.01, 0.01 |
|  | Meditators | 0.06 | 0.02 | 0.00, 0.04 |
| Anger-Hostility | All subjects | 0.04 | 0.01 | 0.00, 0.02 |
|  | Meditators | 0.06 | 0.01 | 0.00, 0.02 |
| Depression | All subjects | 0.21 | 0.01 | 0.00, 0.02 |
|  | Meditators | 0.01 | 0.03 | 0.01, 0.05 |
| Interpersonal Sensitivity | All subjects | <0.01 | 0.02 | 0.01, 0.03 |
|  | Meditators | 0.02 | 0.02 | 0.01, 0.04 |
| Obsessive Compulsion | All subjects | 0.19 | 0.01 | 0.00, 0.02 |
|  | Meditators | 0.01 | 0.04 | 0.01, 0.06 |
| Paranoid Ideation | All subjects | 0.51 | 0.00 | -0.01, 0.02 |
|  | Meditators | 0.21 | 0.01 | -0.01, 0.03 |
| Phobic Anxiety | All subjects | 0.21 | 0.00 | 0.00, 0.00 |
|  | Meditators | 0.03 | 0.01 | 0.00, 0.01 |
| Psychoticism | All subjects | 0.06 | 0.01 | 0.00, 0.01 |
|  | Meditators | 0.06 | 0.01 | 0.00, 0.02 |
| Somatization | All subjects | 0.16 | -0.01 | -0.01, 0.00 |
|  | Meditators | 0.66 | 0.00 | -0.01, 0.02 |
| Symptom Intensity | All subjects | 0.46 | 0.00 | 0.00, 0.01 |
|  | Meditators | 0.63 | 0.00 | -0.01, 0.02 |
| Total Symptoms | All subjects | 0.02 | 0.71 | 0.25, 1.16 |
|  | Meditators | 0.01 | 1.46 | 0.49, 2.43 |

Note: T1 = time point 1; SCL90 = Symptoms Checklist 90; CI = confidence interval
