# Supplementary material for: Slower respiration rate is associated with higher self-reported well-being after wellness training
Source: Sci Rep. 2023 Sep 24;13:15953. doi: 10.1038/s41598-023-43176-w (PMC10518325; doi:10.1038/s41598-023-43176-w)
Supplement: Supplementary file 4 — Supplementary Table S3. [file 41598_2023_43176_MOESM4_ESM.docx]

Table S3. Detailed statistical results for tests of self-reported well-being.

| Model* (type) | Sample | Contrast | *p* | Effect size | CI |
| --- | --- | --- | --- | --- | --- |
| Delta SCL90 ~ Group  (Pre/ Post; LM) | All MNP | MBSR - WL | 0.01 | 0.06 | 0.02, 0.10 |
|  |  | MBSR - HEP | 0.02 | 0.06 | 0.01, 0.11 |
|  | MNP no asthma | MBSR - WL | 0.05 | 0.05 | -0.01, 0.11 |
|  |  | MBSR - HEP | 0.03 | 0.06 | 0.01, 0.12 |
| T1 SCL90 ~ Group | All MNP | MBSR - WL | 0.54 | -0.03 | -0.11, 0.05 |
|  |  | MBSR - HEP | 0.02 | -0.11 | -0.22, -0.03 |
| Delta SCL90 ~ Group + T1 SCL90 (LM) | All MNP | MBSR - WL | <0.01 | 0.06 | 0.02, 0.10 |
|  |  | MBSR - HEP | 0.03 | 0.06 | 0.01, 0.10 |
| SCL90 ~ Group x Time (LMEM) | All MNP | MBSR - WL x T2 - T1 | 0.04 | 0.06 | 0.00, 0.12 |
|  |  | MBSR - HEP x T2 - T1 | 0.02 | 0.08 | 0.01, 0.14 |
| Delta PWB ~ Group  (Pre/ Post) | All MNP | MBSR - WL | 0.31 | -1.90 | -5.59, 1.78 |
|  |  | MBSR - HEP | 0.31 | -2.27 | -6.67, 2.14 |
|  | MNP no asthma | MBSR - WL | 0.12 | -4.36 | -9.81, 1.10 |
|  |  | MBSR - HEP | 0.22 | -3.32 | -8.69, 2.05 |
| Delta MSC ~ Group (Pre/ Post) | All MNP | MBSR - WL | 0.38 | 0.74 | -0.92, 2.4 |
|  |  | MBSR - HEP | 0.52 | 0.64 | -1.29, 2.6 |
|  | MNP no asthma | MBSR - WL | 0.39 | 0.98 | -1.29, 3.24 |
|  |  | MBSR - HEP | 0.53 | 0.67 | -1.45, 2.79 |
| Delta RR ~ Delta SCL90 (H3) | All MNP | - | 0.37 | 0.01 | 0.00, 0.01 |
|  | MNP no asthma | - | 0.05 | 0.01 | 0.00, 0.02 |
| Delta RR ~ Delta PWB (H3) | All MNP | - | 0.97 | -0.02 | -0.73, 0.70 |
|  | MNP no asthma | - | 0.44 | -0.35 | -1.27, 0.56 |
| Delta RR ~ Delta MSC (H3) | All MNP | - | 0.37 | 0.20 | -0.14, 0.53 |
|  | MNP no asthma | - | 0.02 | 0.57 | 0.15, 0.98 |
|  | MBSR no asthma | - | 0.25 | 0.08 | -0.06, 0.23 |
| Post RR ~ Post SCL90 x Group | All MNP | (MBSR + HEP) - WL x SCL90 | 0.84 | 0.00 | -0.01, 0.01 |
|  | MNP no asthma | (MBSR + HEP) - WL x SCL90 | 0.63 | -0.01 | -0.03, 0.01 |
| Post RR ~ Post PWB x Group | All MNP | (MBSR + HEP) - WL x PWB | 0.74 | 0.39 | -1.88, 2.66 |
|  | MNP no asthma | (MBSR + HEP) - WL x PWB | 0.03 | 2.98 | 0.33, 5.64 |
|  |  | MBSR - WL x PWB | 0.10 | 2.58 | -0.51, 5.67 |
| Post RR ~ Post PWB | All MBSR + HEP | - | 0.09 | -1.00 | -2.14, 0.14 |
|  | MBSR + HEP no asthma | - | 0.03 | -1.83 | -3.50, -0.16 |
|  | MBSR no asthma | - | 0.28 | -1.25 | -3.59, 1.09 |
| Post RR ~ Post MSC x Group | All MNP | (MBSR + HEP) - WL x MSC | 0.94 | 0.03 | -0.70, 0.76 |
|  | MNP no asthma | (MBSR + HEP) - WL x MSC | 0.06 | -0.91 | -1.85, 0.04 |

*All models included covariates for age and sex. Note: CI = confidence interval (of effect size estimate); PWB = Psychological Well-being; H = (confirmatory) hypothesis; SCL90 = Symptoms Checklist 90; MSC = Medical Symptoms Checklist; LM = linear model; LMEM = linear mixed effects model; MNP = meditation=naïve participant; WL = waitlist; HEP = health enhancement program (active control); Pre = pre-training period; Post = post-training period
