# Supplementary material for: Slower respiration rate is associated with higher self-reported well-being after wellness training
Source: Sci Rep. 2023 Sep 24;13:15953. doi: 10.1038/s41598-023-43176-w (PMC10518325; doi:10.1038/s41598-023-43176-w)
Supplement: Supplementary file 5 — Supplementary Table S4. [file 41598_2023_43176_MOESM5_ESM.docx]

Table S4. Detailed statistical results for tests of heart rate (HR).

| Model* (type) | Sample | Contrast | *p* | *b* | CI |
| --- | --- | --- | --- | --- | --- |
| T1 HR ~ Age | All subjects | - | 0.31 | 0.03 | -0.03, 0.10 |
| T1 HR ~ Sex | All subjects | - | 0.13 | 1.16 | -0.36, 2.69 |
| T1 HR ~ Group | All Subjects | Meditator - MNP with asthma | 0.04 | 2.70 | 0.26, 5.14 |
|  |  | Meditator - MNP no asthma | 0.03 | 2.27 | 0.12, 4.42 |
|  | All MNP | MNP no asthma - MNP with asthma | 0.42 | 0.75 | -1.07, 2.56 |
| T1 HR ~ T1 SCL90 | All subjects | - | 0.59 | 0.00 | 0.00, 0.00 |
|  | MNP no asthma | - | 0.23 | 0.00 | 0.00, 0.01 |
|  | Meditators | - | 0.71 | 0.00 | 0.00, 0.01 |
| T1 HR ~ T1 PWB | All subjects | - | 0.59 | -0.13 | -0.44, 0.19 |
|  | MNP no asthma | - | 0.23 | -0.31 | -0.81, 0.20 |
|  | Meditators | - | 0.55 | 0.32 | -0.17, 0.80 |
| T1 HR ~ T1 MSC | All subjects | - | 0.59 | 0.05 | -0.06, 0.16 |
|  | MNP no asthma | - | 0.23 | 0.09 | -0.04, 0.22 |
|  | Meditators | - | 0.55 | -0.07 | -0.24, 0.09 |
| Delta HR ~ Group | All MNP | MBSR - WL | 0.24 | 0.04 | -1.78, 1.86 |
|  |  | MBSR - HEP | 0.97 | -1.23 | -3.28, 0.82 |
|  | No asthma | MBSR - WL | 0.65 | -0.57 | -3.00, 1.87 |
|  |  | MBSR - HEP | 0.32 | -1.14 | -3.42, 1.14 |
| Delta HR ~ Delta SCL90 | All MNP | - | 0.67 | 0.00 | 0.00, 0.00 |
|  | MNP no asthma | - | 0.58 | 0.00 | 0.00, 0.01 |
| Delta HR ~ Delta PWB | All MNP | - | 0.52 | 0.14 | -0.15, 0.43 |
|  | MNP no asthma | - | 0.58 | 0.13 | -0.28, 0.54 |
| Delta HR ~ Delta MSC | All MNP | - | 0.52 | -0.06 | -0.19, 0.06 |
|  | MNP no asthma | - | 0.24 | -0.16 | -0.34, 0.02 |

*All models included covariates for age and sex. Note: CI = confidence interval (of effect size estimate); PWB = Psychological Well-being; H = (confirmatory) hypothesis; SCL90 = Symptoms Checklist 90; MSC = Medical Symptoms Checklist; LM = linear model; LMEM = linear mixed effects model; MNP = meditation=naïve participant; WL = waitlist; HEP = health enhancement program (active control)
