# Supplementary material for: Slower respiration rate is associated with higher self-reported well-being after wellness training
Source: Sci Rep. 2023 Sep 24;13:15953. doi: 10.1038/s41598-023-43176-w (PMC10518325; doi:10.1038/s41598-023-43176-w)
Supplement: Supplementary file 6 — Supplementary Table S5. [file 41598_2023_43176_MOESM6_ESM.docx]

Table S5. Detailed statistical results for tests of heart rate variability (HRV).

| Model* (type) | Sample | Contrast | *p* | *b* | CI |
| --- | --- | --- | --- | --- | --- |
| HRV ~ Age | All subjects | - | <0.01 | -0.03 | -0.03, -0.02 |
| HRV ~ Sex | All subjects | - | <0.01 | -0.30 | -0.49, -0.10 |
| HRV ~ Group | All Subjects | Meditator - MNP with asthma | 0.01 | -0.40 | -0.70, -0.10 |
|  |  | Meditator - MNP no asthma | 0.33 | -0.13 | -0.40, 0.13 |
|  | All MNP | No asthma - With asthma | 0.04 | -0.22 | -0.42, -0.01 |
| HRV ~ SCL90 | All subjects | - | <0.01 | -0.06 | -0.10, -0.03 |
|  | Meditators | - | 0.11 | -0.08 | -0.15, 0.00 |
| HRV ~ PWB | All subjects | - | <0.01 | 7.25 | 3.43, 11.06 |
|  | Meditators | - | 0.29 | 3.86 | -2.08, 9.80 |
| HRV ~ MSC | All subjects | - | 0.18 | -0.90 | -2.21, 0.43 |
|  | Meditators | - | 0.58 | -0.64 | -2.97, 1.70 |
| Delta HRV ~ Group | All MNP | MBSR - WL | 0.93 | 0.01 | -0.26, 0.10 |
|  |  | MBSR - HEP | 0.41 | -0.08 | -0.15, 0.17 |
|  | No asthma | MBSR - WL | 0.81 | -0.03 | -0.23, 0.18 |
|  |  | MBSR - HEP | 0.62 | -0.05 | -0.24, 0.14 |
| Delta HRV ~ Delta SCL90 | All MNP | - | 0.42 | -0.02 | -0.02, 0.02 |
|  | MNP no asthma | - | 0.15 | -0.04 | -0.08, 0.01 |
| Delta HRV ~ Delta PWB | All MNP | - | 0.98 | 0.04 | -3.19, 3.27 |
|  | MNP no asthma | - | 0.87 | -0.34 | -4.44, 3.75 |
| Delta HRV ~ Delta MSC | All MNP | - | 0.42 | -0.80 | -2.20, 0.61 |
|  | MNP no asthma | - | 0.05 | -1.98 | -3.60, -0.37 |
| Delta HRV ~ Delta RR | All MNP | - | 0.010 | -0.04 | -0.07, -0.01 |
|  | MNP no asthma | - | 0.045 | -0.03 | -0.07, 0.00 |

*All models included covariates for age and sex. Note: CI = confidence interval (of effect size estimate); PWB = Psychological Well-being; H = (confirmatory) hypothesis; SCL90 = Symptoms Checklist 90; MSC = Medical Symptoms Checklist; LM = linear model; LMEM = linear mixed effects model; MNP = meditation=naïve participant; WL = waitlist; HEP = health enhancement program (active control); Pre = pre-training period; Post = post-training period; RR = respiration rate
